# Transcriptomic and epigenomic profiling datasets of murine HL-1 cardiomyocytes

**DOI:** 10.1016/j.dib.2026.112810

**Published:** 2026-04-30

**Authors:** Anqi Chen, Yinuo Huang, Han Li, Lin Shi, Shu Zhang

**Affiliations:** a GMU-GIBH Joint School of Life Sciences, Guangdong Provincial Key Laboratory of Protein Modification and Disease, The Guangdong-Hong Kong-Macao Joint Laboratory for Cell Fate Regulation and Diseases, Guangzhou Medical University, Guangzhou 511436, China; b Information and Data Management Center, Guangzhou Medical University, Guangzhou 511436, China; c Guangdong Provincial Key Laboratory of Major Obstetric Diseases, Guangdong Provincial Clinical Research Center for Obstetrics and Gynecology, The Third Affiliated Hospital of Guangzhou Medical University, Guangzhou 510150, China

**Keywords:** RNA-seq, ChIP-seq, H3K4me3, H3K27ac, CTCF, Murine cardiomyocyte, HL-1

## Abstract

Epigenetic features, such as histone modifications and higher-order genome organization, are fundamental determinants of cell-specific gene expression. However, while the HL-1 cell line is a widely utilized model for cardiac research, a comprehensive and integrated epigenomic reference for this line remains scarce in public databases. Our dataset comprises multi-omic profiles including H3K4me3, H3K27ac, and CTCF ChIP-seq together with matched RNA-seq data, where H3K4me3, H3K27ac, and CTCF are widely recognized markers of active promoters, distal enhancers, and chromatin architectural boundaries, respectively. The data provide high-quality sequencing reads and well-aligned genomic profiles, which serve as a valuable resource for investigating epigenetic regulation and gene expression. This collection facilitates the identification of regulatory elements and supports the integrative modeling of transcriptional networks in cardiomyocyte models and cardiovascular diseases.

Specifications Table

**Table:** 

Subject	Biology
Specific subject area	Epigenetics, Transcriptomics, Cardiovascular research
Type of data	Table, Raw, Processed, Bulk mRNA-seq, ChIP-seq
Data collection	Total RNA was extracted from HL-1 cells using TRIzol reagent according to the manufacturer’s instructions. RNA-seq libraries were prepared using the Fast RNA-Seq Library Prep Kit V2 (Novogene, Beijing, China) and sequenced on an Illumina platform (NovaSeq X Plus) to generate 150 bp paired-end reads. Raw reads were processed using fastp (v1.0.1) and quality-checked with FastQC (v0.12.1). Clean RNA-seq reads were aligned to the reference genome using STAR (v2.7.11b) with default parameters. For chromatin immunoprecipitation, cells were crosslinked with 1% paraformaldehyde (PFA, Sigma-Aldrich, F8775) in phosphate-buffered saline (PBS), and chromatin was isolated and fragmented by sonication. Immunoprecipitation was performed using antibodies (anti-H3K4me3: ab8580; anti-H3K27ac: ab4729; anti-CTCF: CST3418). ChIP-seq libraries were constructed using the NEB Next Ultra II DNA Library Prep Kit and sequenced on an Illumina platform (NovaSeq X Plus). ChIP-seq reads were processed with fastp and aligned using BWA (v0.7.19-r1273) with default parameters.
Data source location	Institution: GMU-GIBH Joint School of Life Sciences, Guangzhou Medical University City/Town/Region: Guangzhou Country: China
Data accessibility	Repository Name: National Genomics Data Center (NGDC) Data identification number: CRA038456 Direct URL to data: https://ngdc.cncb.ac.cn/gsa/browse/CRA038456/CRR2707778 https://ngdc.cncb.ac.cn/gsa/browse/CRA038456/CRR2707779 https://ngdc.cncb.ac.cn/gsa/browse/CRA038456/CRR2707780 https://ngdc.cncb.ac.cn/gsa/browse/CRA038456/CRR2707781 https://ngdc.cncb.ac.cn/gsa/browse/CRA038456/CRR2707782 https://ngdc.cncb.ac.cn/gsa/browse/CRA038456/CRR2707783 https://ngdc.cncb.ac.cn/gsa/browse/CRA038456/CRR2707784 https://ngdc.cncb.ac.cn/gsa/browse/CRA038456/CRR2707785 https://ngdc.cncb.ac.cn/gsa/browse/CRA038456/CRR2707786
Related research article	None

## 1 Value of the Data

• The H3K27ac dataset facilitates the identification of active enhancers and the annotation of functional regulatory elements in cardiomyocytes.
• The CTCF genomic distribution provides a resource for investigating the three-dimensional (3D) chromatin organization in cardiomyocytes.
• The integration of ChIP-seq profiles (H3K27ac, H3K4me3, and CTCF) with matched RNA-seq data provides a resource for investigating the relationship between epigenetic landscapes and gene expression.
• These data provide an epigenomic map for the HL-1 cells (atrial cardiomyocyte line), which is widely utilized in cardiovascular research.

## 2 Background

Gene expression is orchestrated by a complex interplay of epigenomic mechanisms, encompassing promoter-associated chromatin states, enhancer activities, and higher-order chromatin organization [1,2]. In this study, we present a comprehensive multi-omics dataset derived from HL-1 cells—a proliferative atrial cardiomyocyte line extensively utilized in cardiovascular research [3,4]. By integrating ChIP-seq profiles of pivotal regulatory elements—specifically H3K27ac for active enhancers, H3K4me3 for active promoters, and CTCF for genomic insulation and structural organization—with matched RNA-seq data, this dataset serves as a robust resource for deciphering the intricate relationship between epigenetic landscapes and transcriptional output [5]. These integrated resources offer significant value for epigenomic annotation, regulatory element analysis, and multi-omics data integration within the field of cardiac cell biology.

## 3 Data Description

### 3.1 mRNA-seq data

To characterize the transcriptomic profile of HL-1 cells, total RNA was isolated from three biological replicates. RNA-seq libraries were prepared from the polyadenylated RNA fraction and sequenced on the Illumina NovaSeq X Plus platform using a 150 bp paired-end (PE150) strategy, yielding approximately 63 million paired-end reads in total. The raw sequencing data have been deposited in the National Genomics Data Center (NGDC) under the accession number CRA038456. A summary of the raw data and the reads mapped to the mm10 genome are provided in Table 1, Table 2 respectively.

**Table 1:** Summary of RNA-seq data.

Sample	RNA-seq-rep1	RNA-seq-rep2	RNA-seq-rep3
Total bases	6319,856,400	6350,203,200	6388,615,500
Read1 Q30 bases rate	96.07%	96.11%	96.17%
Read2 Q30 bases rate	95.56%	95.66%	95.74%

Q30: Percentage of bases with quality score (Qphred) > 30.

**Table 2:** Summary of RNA-seq reads mapping to reference genome.

Sample	RNA-seq-rep1	RNA-seq-rep2	RNA-seq-rep3
Total read pairs	21,066,188	21,167,344	21,295,385
Mapped read pairs rate	95.31%	95.63%	95.75%
Unique mapped read pairs rate	91.10%	91.35%	91.53%

### 3.2 ChIP-seq data

To investigate the genome-wide binding landscapes of CTCF and two histone modifications (H3K4me3 and H3K27ac) in HL-1 cells, chromatin immunoprecipitation followed by high-throughput sequencing (ChIP-seq) was conducted. The libraries were sequenced on the Illumina NovaSeq X Plus platform using a 150 bp paired-end (PE150) strategy. A total of 160 million paired-end raw reads were generated. These raw sequencing data have been deposited in the National Genomics Data Center (NGDC) under the accession number CRA038456. The overall sequence quality and the detailed mapping results to the mm10 reference genome are summarized in Table 3, Table 4 respectively.

**Table 3:** Summary of ChIP-seq data.

Sample	H3K4me3-rep1	H3K4me3-rep2	H3K27ac-rep1	H3K27ac-rep2	CTCF-rep1	CTCF-rep2
Total bases	8281,919,100	7418,105,700	7209,420,000	6708,142,800	6452,319,600	6794,653,200
Read1 Q30 bases rate	95.75%	95.65%	94.07%	94.47%	93.84%	95.10%
Read2 Q30 bases rate	93.86%	94.28%	93.04%	94.12%	93.08%	94.02%

Q30: Percentage of bases with quality score (Qphred) > 30.

**Table 4:** Summary of ChIP-seq reads mapping to reference genome.

Sample	H3K4me3-rep1	H3K4me3-rep2	H3K27ac-rep1	H3K27ac-rep2	CTCF-rep1	CTCF-rep2
Total read pairs	27,606,397	24,727,019	24,031,400	22,360,476	21,507,732	22,648,844
Mapped rate	99.99%	99.64%	99.99%	99.68%	99.99%	99.50%
Paired Mapped rate	95.09%	92.26%	74.15%	84.81%	77.11%	85.24%

### 3.3 Integrative visualization of transcriptomic and epigenomic landscapes

**Fig. 1:**
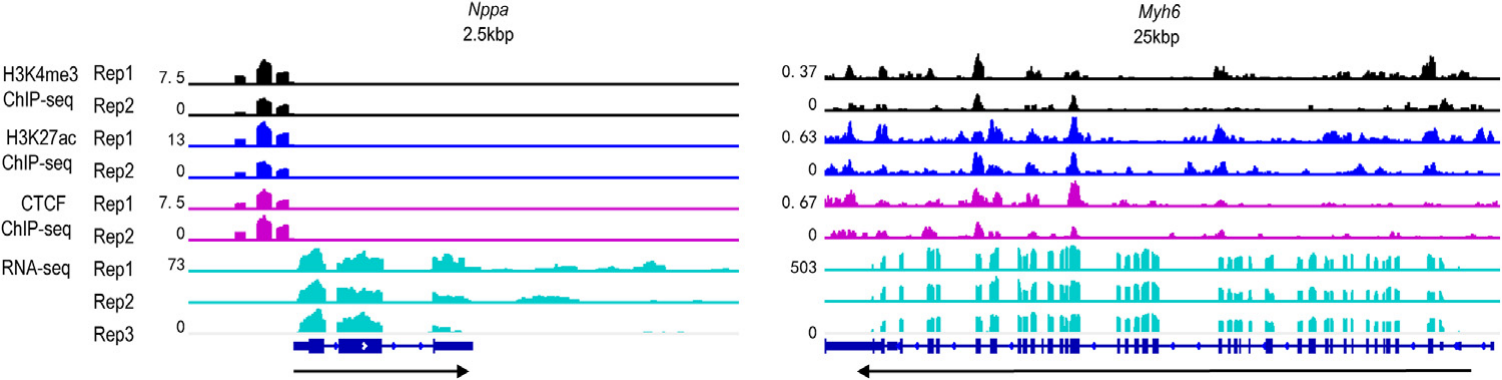
Integrative genomic landscapes of RNA-seq and ChIP-seq profiles in HL-1 cells. Representative genome browser tracks display the signal distribution of transcript abundance (RNA-seq) and epigenetic enrichment (H3K4me3, H3K27ac, and CTCF) at two classical cardiac marker loci: Nppa (left) and Myh6 (right). The RNA-seq signal is represented as normalized read counts in RPKM (Reads Per Kilobase per Million mapped reads), while ChIP-seq profiles for histone modifications and CTCF are normalized using CPM (Counts Per Million mapped reads). The tracks demonstrate high signal-to-noise ratios and consistent co-localization of active epigenetic markers (H3K4me3 and H3K27ac) and CTCF occupancy with transcriptional activity in HL-1 cardiomyocytes.

To provide a visual assessment of the sequencing data, an integrative visualization of RNA-seq and ChIP-seq (H3K4me3, H3K27ac, and CTCF) tracks was performed. Fig. 1 presents representative genomic tracks at the cardiac-related loci *Nppa* and *Myh6*. The distribution of enrichment signals across these datasets shows a clear signal-to-noise ratio, with active epigenetic marks (H3K4me3 and H3K27ac) and CTCF occupancy correlating with the transcriptional activity identified in the RNA-seq data. These profiles demonstrate the consistency between histone modifications, CTCF binding, and gene expression in HL-1 cardiomyocytes.

## 4 Experimental Design, Materials and Methods

### 4.1 Cell culture

HL-1 cells were obtained from Shanghai Fuheng Biotechnology Co., Ltd. HL-1 cells were cultured in Claycomb medium supplemented with 10% FBS, 0.1 mM norepinephrine, and 2 mM l-glutamine. Cells were passaged at a 1:3 ratio upon reaching 100% confluency and grown under 5% CO₂.

### 4.2 RNA extraction and RNA-seq library preparation

Total RNA was extracted from HL-1 cells using TRIzol reagent according to the manufacturer’s instructions. RNA concentration and purity were measured using a NanoDrop spectrophotometer. RNA integrity was assessed using an Agilent 2100 Bioanalyzer. RNA-seq libraries were constructed using the Fast RNA-Seq Library Prep Kit V2 (Novogene, Beijing, China) following the manufacturer’s protocol. The libraries were sequenced on an Illumina platform to generate paired-end reads.

### 4.3 Chromatin immunoprecipitation

HL-1 cells cultured to approximately 80% confluence in 15-cm dishes were crosslinked with 1% paraformaldehyde (PFA, Sigma-Aldrich, F8775) in PBS for 10 min at room temperature and quenched with glycine. Cells were then harvested, and chromatin was isolated. The chromatin was sheared by sonication to generate DNA fragments of appropriate size for immunoprecipitation. Sheared chromatin was incubated overnight at 4 °C with antibodies against the target proteins, followed by incubation with Protein A/G magnetic beads to capture immune complexes. The antibodies used were anti-Histone H3 (tri-methyl K4) (ab8580), anti-acetyl-Histone H3 (Lys27) (ab4729), and anti-CTCF (D31H2) (CST #3418). After immunoprecipitation, crosslinks were reversed, and proteins were digested with proteinase K (New England Biolabs, P8107S). ChIP DNA was subsequently purified and used for library preparation and high-throughput sequencing.

### 4.4 ChIP-seq library preparation

ChIP DNA was processed using the NEBNext Ultra II DNA Library Prep Kit for Illumina (New England Biolabs) following the manufacturer’s protocol. Briefly, DNA fragments were end-repaired and A-tailed prior to adaptor ligation. Ligated DNA was purified and PCR-amplified to enrich adaptor-ligated fragments. Library size distribution and quality were evaluated using an Agilent 2100 Bioanalyzer. Sequencing was performed on an Illumina platform to generate high-quality reads.

### 4.5 Data analysis

The total bases and basic sequencing statistics were calculated using fastp (v1.0.1) [6]. The quality of raw sequencing reads was evaluated using FastQC (v0.12.1). RNA-seq reads were aligned using STAR (v2.7.11b) with default parameters [7]. ChIP-seq reads were aligned using BWA (v0.7.19-r1273) with default parameters [8].

## Limitations

None

## Ethics Statement

The authors have read and follow the ethical requirements for publication in Data in Brief and confirming that the current work does not involve human subjects, animal experiments, or any data collected from social media platforms.

## Credit Author Statement

**Anqi Chen:** Investigation, Methodology, Writing-Original draft. **Yinuo Huang:** Investigation, Methodology. **Han Li:** Formal analysis. **Lin Shi:** Software. **Shu Zhang:** Supervision, Funding acquisition, Writing-Original draft, Writing-Reviewing and Editing.

## Data Availability

NGDCTranscriptomic and epigenomic profiling datasets of murine HL-1 cardiomyocytes (Original data).
